# The Homœopathic Materia Medica; Its Uses and Its Imperfections

**Published:** 1888-11

**Authors:** 


### HOMŒOPATHIC PHYSICIAN,

A MONTHLY JOURNAL OF

HOMŒOPATHIC MATERIA MEDICA AND CLINICAL MEDICINE.

"If our school ever give up the strict inductive method of Hahnemann, we are lost, and deserve only to be mentioned as a caricature in the history of medicine."—CONSTANTINE HERING.

Vol. VIII. NOVEMBER, 1888. No. 11.

### THE HOMŒOPATHIC MATERIA MEDICA; ITS USES AND ITS IMPERFECTIONS.

Ever since the earliest editions of Hahnemann's *Materia Medica Pura* were published, there have been physicians who have continually cried out against its assumed imperfections and errors. The homœopathic materia medica has never been considered perfect, nor has it ever been claimed to be free from errors; nor will it ever be perfect or entirely free from errors, unless, indeed, it find an *infallible* being to create it. Everything that has yet come from the hand of *man* is imperfect, and will doubtless be always so. Considered as a *human* production, the homœopathic materia medica is about as perfect and as free from error as most productions of fallible man, considering its age, noting the comparative few who have labored upon it, observing the many difficulties which surround such work. When these things are remembered, then one is amazed at the state of comparative perfection the homœopathic materia medica *has* attained.

In considering the value of a materia medica, it must be judged according to the purpose for which it is to be used; it will be readily seen that it may be almost perfect for one purpose and entirely useless for another. The materia medica of the old school may be a very useful work when used according to their theories, but it is certainly useless when judged by the homœopathic practice. The same thing may be true if the homœopathic materia medica be used according to the theories of the old school. For example, we can see no guide in our pathogenesis of Opium for giving a hypodermic injection of Morphia for a neuralgia, say; nor would the allopath find any guide in his study of Opium for its use in, say, constipation. The allopath prescribes, in the main, for diseases, or pathological states; the homœopathic physician prescribes for the patient alone, for the symptoms of the individual before him. The practice of each school being so radically different, their therapeutics must also be different; hence he who tries to prescribe the materia medica of one school according to the practice of the other school is sure to find that materia medica imperfect and useless. As we have remarked, there never has been a time in the history of our school when we have been free from harsh critics of our materia medica. These critics have declared the homœopathic materia medica to be little less than a collection of fanciful falsehoods. These critics have used this materia medica, they say, and found it faulty; but how have they used it? Have they not, to a man, used it in an entirely different method from which it is intended to be used? Have they not, to a man, tried to practice a rude kind of allopathy with this homœopathic materia medica? Are their failures to be at all wondered at? Would not success with such methods be more surprising? None of these critics would find the materia medica faulty if they could do good cure-work with it; few or none ever complain of an instrument which serves them well. These gentlemen have, as a rule, been trying to practice allopathy with the homœopathic materia medica, and they have found they could not do it successfully; hence they would revise it so they can better attempt this anomalous feat.

The homœopathic materia medica was never intended to be a treatise upon pathology or physiology or botany, but merely the simple record of the pathogenetic and clinical effects of drugs. This record was created to be used in curing the sick according to the law of the similars, and for nothing else. So long as it enables physicians to do this successfully it is a success; when it fails to be the medium of curing the sick, then it will be a failure. Its value can be judged by no other test.

Those who claim to have faith and confidence in the accuracy of this materia medica also say they can and do make wonderful cures with it; on the other hand, those who declare this materia medica to be erroneous and unreliable are those who not only disclaim success for themselves, but go so far as to deny that others are successful with it. Which gives the more reliable testimony?

Now let us briefly review a few of the items of evidence in favor of the truthfulness of the homœopathic materia medica. It has long been the established rule in evidence that the unimpeached testimony of two or more witnesses is sufficient to prove a case. Many a man has been convicted on less testimony than that we are able to adduce in favor of the accuracy of our materia medica. We claim its accuracy and reliability are proven by—

1. *The symptoms recorded by the provers were mostly new to them; had never been observed before taking the drug.* In Allen's *Encyclopedia* we find this frequently commented upon by the various provers. A sensation that begins as soon as one commences to take an unknown drug and ceases almost as soon as that drug is stopped, supposing also that the sensation had never been noticed before, cannot fairly be ascribed to any other influence than that of the drug taken.

2. *The repetition of similar symptoms in different provers.* Our drugs have been proven upon many different persons. In most cases the name of the drug taken is not known to the prover nor the results of previous provings, yet we find symptoms repeated over and over again in the experiences of different provers. A most noted case of this kind is the now celebrated Austrian re-proving of Natrum muriaticum, which was undertaken by skeptics attempting to show the falsity of Hahnemann's work. These skeptics were convinced against their own avowed prejudices.

3. *The similarity existing between the symptoms of related drugs.* One of the clearest proofs we have of the accuracy of our provings is to be found in the strong likeness existing between the pathogenesis of drugs which are related chemically or botanically. To observe this compare the provings of Bromine, Chlorine, and Iodine; or those of Crotalus, Elaps, Lachesis, etc.; or those of Rhus-tox., Rhus-rad., and Rhus-ven. So also in those compounds in which Phosphorus or Sulphur enter, there will be found throughout their provings numerous traces of Phosphorus or Sulphur, as the case may be; in the provings of the Natrum salts a great similarity exists. This list of examples might be indefinitely extended, but enough cases of similarity have been given here to show the force of the argument.

4. *Clinical confirmation of pathogenetic symptoms.* There can be scarcely any doubt as to the reliability of symptoms which have been produced upon a healthy organism and cured in a sick person; this has been done with the larger portion of the symptoms of our materia medica.

5. *Judged as a whole, the accuracy of the homœopathic materia medica is proven by the clinical results it has achieved.* This materia medica has proven itself to be so useful that the old school has in part adopted it; has attempted to make one somewhat after the same pattern. It is to this materia medica that Homœopathy owes its reputation; it is this materia medica which has enabled physicians to cure many diseases which had hitherto been pronounced incurable; it is this materia medica which has enabled physicians to greatly lower the death-rate in all diseases. Are not these witnesses worthy of credence?

Have the opponents of the homœopathic materia medica ever brought forward any evidence in favor of their views? Do not their attacks upon it consist entirely of unsupported declarations, which are valueless?

